# List of Accidents Admitted into the Pennsylvania Hospital, from January 24th to February 7th, 1838

**Published:** 1838-02-14

**Authors:** 


*List of Accidents admitted into the Pennsylvania Hospital, from January 24th to February 7th*, 1838.

One sprained ankle in a woman. One wound of the face, since discharged, cured. An extensive superficial burn of the back, in a female,—dressed with Goulard's cerate. An incised wound on the inner side of the patella.

